# Validation of a French version of the DERS-SF and creation of a super short form (DERS-F SSF) for measuring emotion regulation difficulties

**DOI:** 10.1038/s41598-025-24281-4

**Published:** 2025-11-19

**Authors:** Simon Thuillard, Noémie Augsburger, Sofia Batziou, Mara Bruni, Laura Fatio, Nahuel Martinez, Elise Mengin, Francesco Ruinato, Livia Sacchi, Natalia Salamon, Mathilde Savary, Elena Trentini, Elise Dan-Glauser

**Affiliations:** Institute of Psychology, University of Lausanne, Bâtiment Géopolis, Quartier UNIL-Mouline, 1015 Lausanne, Switzerland

**Keywords:** Scale validation, Super-short instrument, Emotion regulation difficulties, Psychology, Human behaviour

## Abstract

Difficulties in regulating emotions, i.e., struggling to manage emotions and pursuing goals despite emotions, is a predominant symptom in many diagnoses. In the general population, this variable can be measured with rather comprehensive questionnaires. This is the case of the Difficulties in Emotion Regulation Scale (DERS, 36 items), resulting in a total score and six different sub-scores. In most cases, however, the length and complexity of scale long versions compromise valid recordings. This is the reason why shorter versions of the DERS are available, such as the DERS-SF (18 items). However, when dealing with specific populations, and in particular clinical ones, 18 items remain burdensome. The creation of a Super Short Form seemed necessary in the field. With the present work, we aimed at, first, validating the French translation of the DERS-SF, a widely used instrument, which still lacks French validation. Second, based on an extensive database gathered in our lab, we propose a DERS Super-Short-Form composed of 6 items, each reflecting a difficulty in emotion regulation. Data of 3944 participants (76% female), who completed either the 36-item or the 18-item form of the French DERS were analyzed. Results support the validity and reliability of the French version of the DERS-SF (Cronbach α > 0.84 for the total score and congruence index, Tucker’s ϕ > 0.99). The Super-Short Form demonstrated satisfactory reliability. External validity was confirmed, above and beyond what could be seen with longer scales, enriching the understanding of the various dimensions of the difficulties in emotion regulation.

**Supplementary Information:**

The online version contains supplementary material available at 10.1038/s41598-025-24281-4.

## Introduction

Emotions are central to human experience, and exert a powerful influence on how we perceive and interact with the world, by shaping our perceptions, thoughts, behaviors, and interactions^1^. Emotions are not fixed, immutable experiences, but rather could be (and nearly always are) modulated by the person experiencing them^2^. This capacity is captured by the concept of emotion regulation, a dynamic process that involves the modulation, expression, and understanding of one’s emotions^3^.

Emotion regulation is a multifaceted construct that operates across cognitive, physiological, and behavioral levels. It refers to the processes by which “individuals influence which emotions they have, when they have them, and how they experience and express these emotions” (Gross, 1998, p. 235), allowing individuals to manage emotional responses to various situations and challenges. Effective emotion regulation supports adaptation, enhances social functioning, and promotes mental health and well-being^4–6^. Individuals with effective emotion regulation skills are better equipped to cope with stress, regulate mood, and navigate social interactions successfully. Research consistently links effective regulation to positive outcomes, while difficulties are associated with various forms of psychopathology^7–9^. Beyond mere associations, emotion regulation is thought as being determinant in the onset and maintenance of various mental conditions such as substance use, mood and anxiety disorders, PTSD, and personality disorders^10–15^. Understanding emotion regulation is thus critical not only for fostering well-being and resilience, but also for identifying mechanisms to counteract poor mental health and social dysfunction.

Poor emotion regulation is tightly linked to emotion regulation difficulties, which are involved in many psychopathologies^9^, potentially stemming from a disruption in the acquisition of regulatory skills due to adverse childhood experiences^16,17^. Such difficulties can impair social interactions, academic and occupational performance, and overall quality of life^18,19^. To better contextualize such challenges, Gratz and Roemer^18^ proposed a taxonomy comprising six distinct dimensions of emotion regulation difficulties: (1) Nonacceptance of emotional response : referring to resistance or aversion to experiencing and accepting unpleasant emotions, which hinders the use of adaptive emotion regulation strategies see also^20^. (2) Difficulties in adopting goal-directed behaviors : referring to the difficulties of maintaining focus on purposeful actions during emotional distress, impairing performance. (3) Difficulties in controlling impulsive behaviors : occurring when individuals struggle to manage emotionally triggered impulsive behaviors. It differs from the concept of “Impulsivity”, which includes a broader range of impulsive tendencies that may or may not be influenced by emotional states. (4) Lack of emotional awareness : referring to the difficulty in recognizing and identifying one’s emotions, limiting the ability to understand and regulate emotional experiences, see also^21^. (5) Limited access to emotion regulation strategies : referring to individuals having fewer available strategies for effectively managing their emotions, resulting in difficulties in coping with emotional distress and regulating their mood. It includes the perseverance on one single (often maladaptive) strategy, no matter the context. (6) Lack of emotional clarity : Very similar to lack of emotional awareness, lack of emotional clarity refers to the difficulties in understanding the differences between emotional states. Beyond awareness, this refers to confusion or uncertainty about the nature and causes of one’s emotions and the ability to label them precisely, see also^22^.

This taxonomy offers a comprehensive framework for capturing the key facets of emotion regulation difficulties. Its adoption has enabled the development of more targeted interventions and advanced our understanding of how specific difficulties relate to particular disorders and impairments in psychological functioning. Achieving these goals, however, requires reliable and valid measurement tools. As such, there is increasing demand for robust instruments that can assess emotion regulation difficulties across populations and settings, for both clinical and research purposes. To meet the need for reliable and valid measure of emotion regulation difficulties, Gratz and Roemer developed the Difficulties in Emotion Regulation Scale, the DERS^18^. The DERS has become a widely used and comprehensive self-report tool, designed to assess the six dimensions of emotion regulation difficulties. It comprises 36 items (6 to 8 per dimension), each rated on a 5-point Likert scale from 1 (almost never) to 5 (almost always), reflecting the general frequency of experienced difficulties. Higher scores indicate greater difficulties in emotion regulation^18^. The DERS has demonstrated strong psychometric properties and has been validated across diverse populations^23–32^, making it a standard instrument in both research and clinical contexts.

Despite its widespread use, the DERS’s length can be burdensome—especially in time-constrained contexts or when administered alongside other tools^32–34^. It also poses challenges for participants with limited attention, or cognitive resources^35^. To address this, shorter versions of the DERS have been developed and validated, such as the DERS-18^36^ and the DERS-16^37^. All shorter versions were developed by selecting items from the original scale that show strong psychometric properties and represent each dimension. These abbreviated forms retain the reliability of the full version and have been validated in diverse samples, including adolescents, adults, and clinical populations, demonstrating their utility across different age groups and settings^36–41^. Among these short forms, the DERS-Short Form, the DERS-SF^42^, stands out. Indeed, in addition to reliable and stable psychometric properties^43–45^, and compared for example to the DERS-16, the DERS-SF possesses three items per subscale, allowing for scoring each difficulty separately. Moreover, and as an advantage over the DERS-18, it shows stronger invariance and has been recommended as the most reliable among the short forms^44^.

Validating emotion regulation scales in different languages is essential to ensure their reliability and cultural relevance. Indeed, since emotion regulation is shaped by cultural norms, it can influence how emotions are experienced and expressed^46^ and a simple translation may not be efficient to capture this variability. The original DERS with 36 items have been translated and validated in multiple languages (e.g., Arabic, Malay, Ecuadorian, Italian, Persian, Indonesian)^47–51^, including in French, where there exists two validations, one coming from a Swiss-sample validation study^52^ and one coming from a French-Canadian-sample validation study^53^. The shortened versions of the DERS have also been validated in various languages but there is currently a dearth of validated short versions of the scale in French, despite the language being spoken by over 300 million people. This lack limits both clinical use and cross-cultural research, creating a notable gap in the literature.

Beyond cultural adaptation, tailoring measurement tools to specific settings is equally crucial. The length of the original 36-item DERS led to the development of shorter versions (16 or 18 items), which offer benefits such as efficiency, cost-effectiveness, and reduced participant fatigue^54,55^. When developed and validated properly, they can provide reliable and valid measurements that are comparable to longer tests, making them an invaluable tool in both research and applied contexts^56^. However, in some settings, or when examining complex multivariate questions, 16 or 18 items may already be too many, in particular for people with attention deficits or other specific conditions such as addiction disorders, see^57–59^. These populations may struggle to complete lengthy questionnaires, underscoring the need for briefer but still robust measures. Two previous works attempted to outline a even shorter version of the DERS than 16 or 18 items. The first one was developed by Espirito-Santo and colleagues^60^, who used the DERS-16 in Portuguese older adults (*N* = 302), extracted 8 items yielding a total score and four subscale scores (Clarity, Impulse, Goals, and Non-acceptance). The second very short version of the DERS was developed by Penner and colleagues^61^, also comprising eight items. From the 36-item English version, authors limited the original pool to 27 items, excluding *de facto* the clarity subscale due to the removal of items that lack a pure regulation preface wording. *N* = 700 students were used to validate the version and additional 619 adolescents with clinical diagnoses were recruited for further validation. The resulting eight-item version gives a total score as the only outcome. These short forms show promise for clinical screening^27,38,62,63^. We believe, however, that the current short forms of the DERS detailed above do not fully meet the needs of the field, particularly in research settings, where refinement of the different difficulties highlighted in Gratz and Roemer’s taxonomy would inform on more subtle mechanisms leading to detrimental outcomes.

The present work stems from the established link between emotion regulation difficulties and mental health, highlighting the need for valid, efficient tools. Although several short DERS forms exist, only the full 36-item version has been validated in French, which limits its usability in time-sensitive clinical and research contexts. To address this, we aimed to develop a validated ultra-short French version that maintains the scale’s conceptual depth while improving feasibility. Our study had hence two goals. First, using a large French-speaking sample, we validated the DERS-SF based on the published English items and the existing French translation of the full DERS^52^. Second, to support research in resource-constrained settings (e.g., Ecological Momentary Assessment or large-scale surveys), we developed and validated a Super-Short Form tailored to young adults. Importantly, our goal was to preserve differentiation across the six key emotion regulation difficulties by retaining one representative item per dimension. This methodological article details the validation process of the DERS-SF in French, including its psychometric properties and utility in assessing emotion regulation difficulties in a French-speaking population. By offering a validated French short form, the study supports the international use of this key instrument and encourages cross-cultural research on emotion regulation. In addition, we aim to provide a super-short form of the DERS, obtained from a large adult sample (approximately 4000, aged 18 to 45), which is designed to measure difficulties in emotion regulation across its six dimensions. For both objectives, we took advantage of data recorded in our lab in previous years. In this database, we benefitted from participants having filled the 36-item version, from which we extracted the corresponding DERS-SF 18 items (called the “extracted version”) as well as participants having filled the (not yet validated) 18-item French version of the DERS-SF (called the “standalone version”).

For the present work we had the following hypotheses:

### H1

The French version of the DERS-SF (F-DERS-SF) will retain similar properties in terms of structure as the DERS 36-item version, both in its extracted version and in the standalone version.

### H2

The F-DERS-SF will have the same external validity as the corresponding DERS-F with regard to general positive and negative affects, depressive and anxiety symptoms, and alexithymia levels.

### H3

The extraction of a six-item Super Short Version of the French DERS will lead to items forming a total score. The respective six items will inform individually on each original subscale scores, having a significant relationship with the corresponding DERS-36 and DERS-SF sub-scores.

### H4

The Super Short Version of the French DERS will retain satisfactory external validity for general positive and negative affects, depressive and anxiety symptoms, and alexithymia levels.

## Results

### Descriptive scores and gender invariance

Table 1 below shows the values of the total scores and dimension scores obtained for emotion regulation difficulties measures with the different instruments and dataset variants. Note that scores of the DERS-F SSF have already been included for reference. Details on the validity of the measures are given later in the Result section.

**Table 1:** Difficulties in emotion regulation total and dimension descriptives for each dataset.

	DERS-F	Standalone DERS-SF	Extracted DERS-SF	DERS-F SSF
Total Score	2.54 (0.64, 1.06–4.69)	2.60 (0.63, 1.06–4.61)	2.44 (0.66, 1–4.67)	2.38 (0.70, 1–4.67)
Non-acceptance	2.29 (1.00, 1–5)	2.33 (1.10, 1–5)	2.16 (1.11, 1–5)	2.09 (1.21, 1–5)
Goal	3.31 (0.99, 1–5)	3.37 (1.14, 1–5)	3.35 (1.07, 1–5)	3.49 (1.15, 1–5)
Impulse	2.24 (0.94, 1–5)	2.28 (1.10, 1–5)	2.04 (1.05, 1–5)	1.94 (1.15, 1–5)
Awareness	2.54 (0.80, 1–5)	2.48 (0.88, 1–5)	2.38 (0.86, 1–5)	2.30 (1.08, 1–5)
Strategies	2.58 (0.90, 1–5)	2.67 (1.03, 1–5)	2.51 (1.00, 1–5)	2.22 (1.18, 1–5)
Clarity	2.35 (0.79, 1–5)	2.47 (0.88, 1–5)	2.19 (0.83, 1–5)	2.25 (1.09, 1–5)

Mean scores are indicated, followed by standard deviation in brackets. Ranges (min-max) are then indicated. DERS-F is 36 items, Standalone and Extracted SF are 18 items each, the SSF is 6 items total. For the DERS-F SSF, only the Unweighted total score is reported, Weighted values are M = 2.35, SD = 0.71, with a range of 1 to 4.69.

Table 2 shows the values for each difficulty, and, when applicable, total scores of external validity measures (PANAS, PHQ-4 and TAS).

**Table 2:** Scores of the external validity measures.

	Mean (SD)	Range
PANAS		
Negative scale	2.24 (0.75)	1–5
Positive scale	3.26 (0.71)	1.1–5
PHQ-4		
Anxiety	2.11 (1.65)	0–6
Depression	2.78 (1.84)	0–6
TAS		
Total Score	2.51 (0.55)	1–4.15
Difficulty identifying feelings	2.62 (0.81)	1–5
Difficulty describing feelings	2.98 (0.95)	1–5
Externally-oriented thinking	2.13 (0.54)	1–4.88

Mean scores are indicated, followed by standard deviation in brackets. Ranges (min-max) depend on the scale: PANAS and TAS go from 1 to 5, PHQ-4 scores are measured on a scale from 0 to 6.

Measurement invariance analyses across gender supported configural, metric, and scalar invariance for the 36-item, 18-item standalone, 18-item extracted, and 18-item full versions of the DERS, indicating equivalent functioning for male and female respondents (see Supplementary Table 1 for full fit indices).

### Structural properties and internal reliability of the French version of the F-DERS-SF

The three EFA, performed on the three different samples (*Full Sample*, *Extracted F-DERS-SF*,* Standalone F-DERS-SF)* with number of factors constrained by parallel analyses, all led to 6 factor solutions, with items grouping according to the theoretical expectations. The three 6-factor models all explained similar percentage of total variance: 61.7% for the *Standalone F-DERS-SF*, 63.0% for the *Extracted F-DERS-SF*, and 61.9% for *Full Sample*. Models were all significant, χ^2^_(60)_ = 254–562, all *p* <.001, and model fit indices were satisfactory to excellent (RMSEA = 0.032–0.074.032.074, TLI = 0.90–0.98.90.98). Table 3 shows factor loadings for each item and for the three models. We considered factor loading > 0.70 to be strong, > 0.50 to be moderate, > 0.30 to be weak, > 0.20 to be very weak, and < 0.20 (to be discarded).

**Table 3:** Factor loadings for each subcomponent of the F-DERS-SF for the three sub-samples extracted F-DERS-SF/Full Sample/Standalone F-DERS-SF).

	Factor 1: Non-Acceptance	Factor 2: Impulse	Factor 3: Goals	Factor 4: Awareness	Factor 5: Strategies	Factor 6: Clarity
% Variance	12.5/12.9/11.0	12.5/12.7/11.5	12.1/12.4/12.6	8.0/8.7/6.0	9.1/8.4/11.6	7.7/7.8/8.9
Cumulated % Variance	12.5/12.9/11.0	25.0/25.7/22.5	37.1/38.0/35.1	45.1/46.7/41.1	54.2/55.2/52.8	61.9/63.0/61.7
Item 12	0.96/0.95/0.88					
Item 16	0.92/0.89/0.77					
Item 7	0.78/0.77/0.74					
Item 17		0.95/0.93/0.82				
Item 14		0.86/0.83/0.81				
Item 9		0.81/0.82/0.71				
Item 11			0.90/0.86/0.86			
Item 13			0.87/0.85/0.80			
Item 8			0.79/0.81/0.77			
Item 1				0.84/0.78/0.58		
Item 4				0.77/0.76/0.64		
Item 6				0.46/0.47/0.47		
Item 18					0.82/0.78/0.54	
Item 15					0.71/0.69/0.62	
Item 10					0.57/0.56/0.50	
Item 3						0.94/0.83/0.81
Item 2						0.73/0.81/0.88
Item 5					0.23/0.11/**0.51**	0.31/0.26/0.22

Factor extraction order corresponds to the *Extracted F-DERS-SF* sample, which has the best fit indices. Loadings are shown with the following sequence *Extracted F-DERS-SF*/*Full Sample*/*Standalone F-DERS-SF*. Items list includes only the maximal loadings, in general confirming theoretical factor placement (maximal loadings across Factors). One can see a loading for 1 item with theoretical factor misplacements (item 5 with the *Standalone F-DERS-SF*).

Confirmatory analyses conducted on the three samples confirmed the original structure, χ^2^_(120)_ = 1181–2256, all *p* <.001. Model fit indices are summarized in Table 4. In all three analyses, all items significantly contributed to the latent factor, standardized estimates = 0.20–0.90, all *p* <.001.

**Table 4:** Model fit indices for the CFA conducted on the three samples.

	RMSEA	TLI	CFI
Extracted F-DERS-SF	0.05	0.95	0.96
Full Sample	0.07	0.92	0.94
Standalone F-DERS-SF	0.11	0.79	0.83

*RMSEA *Root Mean Square Error of Approximation, *TLI* Tucker–Lewis Index, *CFI *Comparative Fit Index.

Cronbach α analyses showed very good reliability for the total scale: 0.88 for the *Extracted F-DERS-SF*, 0.87 for the *Full Sample*, and 0.84 for the *Standalone F-DERS-SF*. Removal of Item 5 (the only item with weak to very weak loading factor, see Table 3) did not improve the reliability. We hence decided to retain it because of the large sample size (which reduces the threshold for meaningful loadings) and the theoretically meaningful phrasing of the item. Cronbach α for each subscale demonstrated reliability that, as expected, followed the values provided by the full 36-items scale: Non-Acceptance = 0.84–0.89, Impulse = 0.86–0.89, Goals = 0.87–0.88, Awareness = 0.64–0.72, Strategies = 0.75–0.76, and Clarity = 0.61–0.69. For this latter subscale, removal of Item 5 led to alphas increasing to the 0.77–0.80 level. A particular consideration of this item should thus be made (but see very satisfactory correlation table below).

Mean inter-item correlations gave Pearson’s correlation coefficients of 0.26, *p* =.001 for the *Full Sample*, 0.28, *p* <.001, for the *Extracted F-DERS-SF* and 0.21, *p* =.008, for the *Standalone F-DERS-SF.* Mean inter-item Pearson’s correlation coefficients within subscales showed very high interrelation between items belonging to the same subscale, Non-Acceptance = 0.63–0.73, Impulse = 0.67–0.73, Goals = 0.69–0.71, Awareness = 0.37–0.47, Strategies = 0.50–0.52, and Clarity = 0.34–0.43. Corrected Pearson’s correlations between items and total score and corrected Pearson’s correlations between items and their own subscale scores are given Table 5, all *p*s are significant at the *p* <.001 level with their respective corrected subscale scores, *ps* for the correlations with the total score are all < 0.05, except for Item 6 with the *Standalone F-DERS-SF* (*p* =.31).

**Table 5:** Corrected pearson’s correlation coefficients between each item and its corresponding subscale and total score.

	Factor 1: Non-Acceptance	Factor 2: Impulse	Factor 3: Goals	Factor 4: Awareness	Factor 5: Strategies	Factor 6: Clarity	Total Score
Item 12	0.82/0.80/0.73						0.55/0.54/0.48
Item 16	0.81/0.79/0.73						0.58/0.56/0.52
Item 7	0.72/0.70/0.61						0.52/0.51/0.44
Item 17		0.80/0.80/0.78					0.60/0.60/0.60
Item 14		0.80/0.78/0.71					0.63/0.62/0.57
Item 9		0.75/0.74/0.66					0.63/0.62/0.57
Item 11			0.78/0.78/0.78				0.54/0.51/0.48
Item 13			0.78/0.79/0.79				0.58/0.55/0.49
Item 8			0.72/0.73/0.68				0.56/0.57/0.57
Item 1				0.62/0.58/0.38			0.23/0.20/0.09
Item 4				0.58/0.57/0.48			0.20/0.17/0.08
Item 6				0.40/0.42/0.44			0.04/0.05/**0.03**
Item 18					0.59/0.59/0.58		0.54/0.52/0.46
Item 15					0.61/0.60/0.59		0.64/0.63/0.62
Item 10					0.58/0.57/0.54		0.61/0.60/0.55
Item 3						0.59/0.61/0.58	0.51/0.49/0.38
Item 2						0.54/0.55/0.49	0.48/0.43/0.27
Item 5						0.35/0.33/0.20	0.49/0.48/0.42

Factor extraction order corresponds to Table 3 above, that is the order of the best fit indices. Correlations are shown with the following sequence *Extracted F-DERS-SF*/*Full Sample*/*Standalone F-DERS-SF*. All correlations are significant (all *p* <.05 for correlations with total scores), except the one in bold; all *p* <.001 with the corrected subscale.

Tucker’s ϕ (congruence index) for the two matrices of loadings (the DERS-F 36 items, and the F-DERS-SF) obtained on the constrained EFA (6 factors) on the common 18 items gave excellent results: Tucker’s ϕ = 0.997 for the *Full Sample*; Tucker’s ϕ = 0.991 for the *Standalone F-DERS-SF*; and Tucker’s ϕ = 0.996 for the *Extracted F-DERS-SF*.

Afterwards, we performed standard Pearson’s correlation analyses. First, the total score between the 36-item version and the translated 18-item version (calculated from the *Extracted F-DERS-SF* items) showed very high association, r_(3074)_ = 0.98, *p* <.001. The same analyses on the different subscales also gave excellent results: r_(3074)_ = 0.96 for the Non-Acceptance subscale, r_(3074)_ = 0.95 for the Impulse subscale, r_(3074)_ = 0.97 for the Goal subscale, r_(3074)_ = 0.93 for the Awareness subscale, 0.94 for the Strategies subscale, and 0.93 for the Clarity subscale, all *p* <.001. As a final analysis for this section, external validity was checked against scores of general affect, as well as anxiety, depression, and alexithymia symptom scores. Results are shown in Table 6.

**Table 6:** Pearson’s correlation coefficients between DERS scores and emotionality and symptom scores.

	Positive Affect (PANAS)	Negative Affect (PANAS)	Anxiety Symptoms (PHQ-4)	Depression Symptoms (PHQ-4)	Alexithymia Identifying (TAS)	Alexithymia Describing (TAS)	Alexithymia External th. (TAS)	Alexithymia Total Score (TAS)
Ders-36 items								
**Total Score**	− 0.20***	0.50***	0.49***	0.49***	0.65***	0.43***	0.12***	0.57***
Non-Acceptance	− 0.08**	0.39***	0.35***	0.37***	0.46***	0.28***	0.02	0.37***
Impulse	− 0.09***	0.44***	0.35***	0.40***	0.47***	0.18***	0.05	0.34***
Goals	− 0.10***	0.33***.	0.27***	0.36***	0.38***	0.18***	− 0.07**	0.25***
Awareness	− 0.20***	0.06*	0.14***	0.04	0.25***	0.42***	0.41***	0.47***
Strategies	− 0.18***	0.49***	0.49***	0.49***	0.52***	0.26***	0	0.38***
Clarity	− 0.20***	0.36***	0.41***	0.35***	0.71***	0.59***	0.19***	0.70***
*Extracted F-DERS-SF*								
**Total Score**	− 0.17***	0.49***	0.47***	0.46***	0.63***	0.42***	0.12***	0.56***
Non-Acceptance	− 0.06*	0.34***	0.31***	0.32***	0.40***	0.27***	0.03	0.33***
Impulse	− 0.04	0.39***	0.28***	0.31***	0.41***	0.17***	0.07**	0.31***
Goals	− 0.09***	0.30***	0.26***	0.34***	0.37***	0.16***	− 0.07**	0.23***
Awareness	− 0.19***	0.05*	0.11***	0	0.23***	0.38***	0.36***	0.43***
Strategies	− 0.16***	0.45***	0.49***	0.46***	0.48***	0.25***	0	0.36***
Clarity	− 0.14***	0.39***	0.41***	0.38***	0.66***	0.51***	0.14***	0.62***
*Full Sample*								
**Total Score**	− 0.19***	0.48***	0.45***	0.45***	0.61***	0.40***	0.11***	0.53***
Non-Acceptance	− 0.07**	0.34***	0.30***	0.32***	0.38***	0.25***	0.02	0.31***
Impulse	− 0.05	0.38***	0.30***	0.34***	0.40***	0.17***	0.07***	0.31***
Goals	− 0.12***	0.29***	0.18***	0.26***	0.35***	0.16***	− 0.07**	0.22***
Awareness	− 0.19***	0.04	0.15***	0.05*	0.19***	0.34***	0.33***	0.38***
Strategies	− 0.18***	0.44***	0.40***	0.39***	0.47***	0.25***	0	0.35***
Clarity	− 0.16***	0.38***	0.43***	0.40***	0.61***	0.47***	0.13***	0.57***
*Standalone F-DERS-SF*								
**Total Score**	− 0.29***	0.44***	0.39***	0.44***	0.46***	0.32***	0.10	0.41***
Non-Acceptance	− 0.08	0.33***	0.28***	0.34***	0.29***	0.17**	− 0.04	0.20***
Impulse	− 0.02	0.36***	0.33***	0.42***	0.33***	0.17**	0.11*	0.28***
Goals	− 0.25***	0.23***	0	0.04	0.27***	0.18***	− 0.06	0.19***
Awareness	− 0.22***	− 0.07	0.25***	0.19***	− 0.02	0.13*	0.21***	0.13*
Strategies	− 0.27***	0.42***	0.16**	0.17**	0.38***	0.21***	0.04	0.29***
Clarity	− 0.27***	0.35***	0.45***	0.43***	0.37***	0.30***	0.11*	0.36***
*DERS-F SSF*								
**Total Score**	− 0.16***	0.44***	0.43***	0.43***	0.61***	0.44***	0.14***	0.56***
Non-Acceptance	− 0.06*	0.30***	0.26***	0.28***	0.36***	0.25***	− 0.02	0.30***
Impulse	− 0.02	0.34***	0.26***	0.29***	0.36***	0.13**	0.03	0.26***
Goals	− 0.08**	0.24***	0.21***	0.30***	0.31***	0.12***	− 0.09***	0.18***
Awareness	− 0.14***	0.04	0.12***	0.02	0.19***	0.35***	0.32***	0.38***
Strategies	− 0.15***	0.39***	0.39***	0.36***	0.40***	0.23***	0.10***	0.35***
Clarity	− 0.11***	0.26***	0.32***	0.29***	0.59***	0.54***	0.15***	0.60***

*p <.05; **p <.01; ***p <.001. For the DERS-F SSF, only the Unweighted total score is reported, Weighted values are identical in terms of impact and significance (bigger differences total 0.02 on the r values).

### Validation of the French super short 6-item version

Table 7 below gives the chosen items for the 6-items F-DERS SSF, both in its French version and in the original English version.

**Table 7:** Items of the 6 items F-DERS SSF with the corresponding English items.

Dimension	Item *N*°	French items	English items
Clarity	5	J’ai des difficultés à donner un sens à mes sentiments	I have difficulty making sense out of my feelings.
Awareness	6	Je fais attention à mes sentiments (r)	I pay attention to how I feel. (r)
Impulse	14	Quand je suis contrarié(e), je deviens incontrôlable	When I’m upset, I become out of control.
Goals	18	Quand je suis contrarié(e), j’ai des difficultés à me concentrer sur d’autres choses	When I’m upset, I have difficulty focusing on other things.
Non Acceptance	25	Quand je suis contrarié(e), je me sens coupable de ressentir une telle émotion	When I’m upset, I feel guilty for feeling that way.
Strategies	28	Quand je suis contrarié(e), je crois qu’il n’y a rien que je puisse faire pour me sentir mieux	When I’m upset, I believe that there is nothing I can do to make myself feel better.

Items N° refers to the DERS-F numbering, r are reversed items.

Descriptives of the *DERS-F SSF* can be found in Table 1. Unfortunately, Cronbach α did not give excellent results but stayed at a moderate level, α = 0.66. Removal of the item corresponding to the Awareness scale resulted in an increase of the alpha to α = 0.70, which then reached acceptable internal consistency.

Mean inter-item Pearson’s correlation gave correlation coefficients of 0.24, *p* <.001. Corrected item-total score (both weighted and unweighted) gave all significant Pearson’s correlations at *p* <.001 significance level: r_(3076)_ = 0.43–0.44 for Clarity, r_(3076)_ = 0.13 for Awareness, r_(3075)_ = 0.47–0.48 for Impulse, r_(3076)_ = 0.37–0.39 for Goals, r_(3076)_ = 0.38–0.39 for Non-Acceptance, and r_(3075)_ = 0.55 for Strategies. First number is with TS_Unweighted and second is with TS_Weighted, only one correlation means they both align to the same r value.

Turning to correlations with the longer versions of the DERS, the scores obtained with the 36-item version (both total and subscales) align in an excellent fashion with the scores of the *DERS-F* SSF. Indeed, 36-items total score correlated to *r* =0.93 with the *DERS-F SSF* (same value for Weighted and Unweighted Total Scores). Subscales aligned also very well, *r* =0.88 for Non-Acceptance and Goals, *r* =0.86 for Impulse, *r* =0.83 for Clarity, *r* =0.81 for Awareness, and *r* =0.80 for Strategies. This means that the sole use of six items could very well reproduce what can be achieved with the 36-item version. Comparisons between the *DERS-F SSF* and the *Full Sample* testing the 18-item version and the *Extracted F-DERS-SF* also gave very satisfactory results. With the *Full Sample* 18-item form, we found *r* =0.94 with the total scores of the *DERS-F SSF* (same value for Weighted and Unweighted Total Scores). Subscales also aligned very well, *r* =0.92 for Non-Acceptance, *r* =0.90 for Goals, *r* =0.89 for Impulse, *r* =0.85 for Clarity, *r* =0.83 for Strategies, and *r* =0.75 for Awareness, all *p* <.001.

Finally, to test H4, we explored the Pearson’s correlations of the *DERS-F SSF* scores and the scores provided by the external validity scales (PANAS, PHQ-4 and TAS-20). These correlations can be found in the last section of Table 6.

## Discussion

The present study had two main objectives. First, we aimed to validate the French version of the DERS-SF using a comprehensive database of French-speaking individuals. Additionally, recognizing the need for a super short version to facilitate research in contexts with limited time, resources, or participants, we sought to validate a French Super-Short Form. We wanted this version to be suitable for a large, predominantly younger adult population. Most importantly, we wanted the Super Short Form to retain the ability to provide specific information on the various dimensions of emotion regulation difficulties identified in the longer original version.

We had four hypotheses. First, we expected that the F-DERS-SF would bear similar properties in terms of structure as the DERS 36-item version. This hypothesis was confirmed with a six-factor satisfactory to excellent model given by the exploratory factor analysis. Confirmatory factor analyses conducted across three samples provided strong to acceptable fit in two cases (*Extracted F-DERS-SF* and Full Sample) but indicated weaker fit in the *Standalone F-DERS-SF*. The observed discrepancy may stem from sample size differences. Importantly, factor loadings remained stable across samples, and additional psychometric analyses (Cronbach α, Tucker’s ϕ) further support the robustness of the scale. Given this evidence, we consider adequate the use of the 18-item version of the F-DERS-SF with the corresponding items of the full version. A few elements did not align with what was expected, such as item 5 not being identified as representing lack of clarity for one of our sub-samples and item 6 not correlating with the total score within the same sample. Given the doubt casted on the two dimensions of awareness and clarity in the past, we will discuss this issue further below. Our second hypothesis stated that the F-DERS-SF would have the same external validity as the corresponding DERS-F for general affects, depressive and anxiety symptoms, and alexithymia levels. Again, this hypothesis was largely confirmed. However, one point needs to be highlighted, which concerns the relationship (or lack thereof) between the TAS subscale “Externally oriented thinking” and several of the subscales of the different versions of the DERS we tested. We think this point also deserves further discussion, detailed below. Our third and fourth hypotheses dealt with the extraction of a six-item Super Short Version of the French DERS. We first assumed that this super short form would lead to items forming the total score and subscale scores having significant relationships with the corresponding DERS-36 and DERS-SF scores. Moreover, we postulated that the DERS-F SSF would retain satisfactory external validity for general affects, depressive and anxiety symptoms, and alexithymia levels. Again, both hypotheses were generally confirmed. Relationships between our shorter and the longer scales was unexpectedly high and showed that six items alone could well reproduce what can be obtained with the 36-item version, both for the general evaluation of the difficulties in emotion regulation and the evaluation of the six distinct difficulties. External validity was excellent too, strictly reproducing the pattern observed with longer instruments in the relationship with emotional symptoms. During the DERS-F SSF validation, however, the Cronbach’s α was lower than expected and revolved around 0.66. Removing a problematic item related to the lack of awareness increased the alpha to the 0.70 threshold. However, we believe that this solution is too simplistic and we hence present below a discussion of this specific result. Together with our reflection on the specific particularities of the Awareness subscale, we suggest to retain the Awareness subscale in the evaluation.

The Awareness and Clarity subscales stand out in these analyses. In several instances, they showed results that deviate from their pure association with other emotional symptoms and emotional difficulties. First of all, we saw in our results that one of the DERS-SF items (item 5 : “I have difficulty making sense out of my feelings”) loaded preferentially on the *Limited access to emotion regulation strategies* subscale, and not on its own factor (*Lack of emotional clarity*). This is peculiar since this item refers to the fact of being puzzled by one’s own emotions. We can hypothesize that being lost in the face of emotions can lead to misinterpreting emotional signals, causing individuals to feel paralyzed in the face of deciding which strategy to implement. It is quite difficult to imagine, however, how any other item may not be fully related to this, and we thus suggest particular caution with this item in future studies. One item of the Awareness subscale also showed (albeit in only one of our samples) a non-significant relationship with the total score. The item (item 6: “I pay attention to how I feel”) thus seems to tap into a concept that is further away from emotion regulation difficulties than the other items. We develop this point further below. When examining the relationship between emotion regulation difficulties and emotional symptoms, we noticed that Awareness is the subscale showing weaker association with different symptoms, in particular with depression and negative affect, and this happens throughout the versions we have tested here.

The psychometric properties of the DERS have been extensively studied in the past, with clear validity but somewhat mixed results regarding its factor structure. Some studies proposed alternative structures, including five-factor models^64,65^, four-factor models^66^ and bi-factor models^67^. The Awareness and Clarity factors have been particularly scrutinized, with some researchers suggesting they could be combined^65,66^. For other researchers, the DERS should be used without the Awareness subscale due to its poor psychometric properties^24,38,39,64^. It is true that excluding the Awareness subscale from the DERS results in better overall scale performance. For instance, models without the Awareness subscale exhibited better fit and internal consistency^44^. It has also been found that the Awareness subscale shows weak or even non-significant associations with various indices of psychopathology, such as anxiety and depression, compared to other subscales which have stronger correlations with these conditions^68,69^. What’s more, one study even found consistent negative correlations between the Awareness subscale and several symptoms, which were not observed with other subscales^70^. In summary, it seems there are recurrent problems with the subscale of Awareness and (to a lesser extent) with the subscale of Clarity, aligning with what we found in the current study. This recurrent pattern calls for deeper theoretical examination, beyond psychometric concerns alone.

Regarding Awareness, one can discuss the origin of the many psychometric problems associated with it. The Awareness subscale may assess a different construct than the other subscales. In fact, while the other subscales measure how individuals respond to emotions, the Awareness subscale measures whether individuals are aware of their emotions. In that sense, it might capture a more basic, upstream process than regulation *per se*. Emotional awareness, while necessary, may not be sufficient for emotion regulation and may not align with the construct of emotion regulation difficulties measured by the other subscales. This distinction may explain why awareness does not behave like a core regulatory difficulty, but rather as a precondition to the implementation of processes on which difficulties may occur. In a sense, we could see Awareness as the foundation of all difficulties, since there can be no successful regulation if the individual is not even aware that there are emotions to regulate. From this perspective, Awareness operates as a gateway rather than a mechanism of regulation itself. This may be why we observe a decreasing inter-correlation between the subscales, but also why the correlation becomes weaker the more we turn to the measurement of the general construct. Another issue is how this subscale is measured. It seems unreliable to us to ask individuals who are not aware of their own emotions whether they feel they are aware of their own emotions. Such metacognitive judgments may be inherently unstable, particularly in clinical populations. If the goal is to focus specifically on Awareness, other, more reliable measures, could be used, ideally containing some implicit measurements, such as the Levels of Emotional Awareness Scale LEAS^21^. If the goal is to stick to self-report measures, the question is whether the Awareness subscale should be used in the total score or whether it should remain as a separate indicator. Based on both psychometric limitations and theoretical considerations, we suggest that the Awareness subscale need to be considered separately from the DERS total score in future applications. Specifically, we recommend reporting a DERS Awareness subscore independently, alongside a revised total score calculated without this subscale, in order to preserve both dimensional integrity and practical interpretability. This approach would allow researchers to preserve the relevance of emotional awareness while acknowledging its conceptual and psychometric specificity. Finally, regarding Clarity, and despite the need to pay attention to item 5 of the DERS-SF, we agree with Ekland and Thompson^71^, who emphasized the central role of emotional clarity in emotional competences. We would hence advocate to maintain Clarity into the measurements, given also that the subscale, as opposed to the Awareness subscale, demonstrated moderate to strong associations with the measured psychopathology symptoms.

While examining the external validity of the F-DERS-SF, the relationship with the subscale “Externally-oriented thinking” from the TAS has attracted attention and deserves to be further elaborated on. This subscale refers to a cognitive style where individuals focus on external events and concrete details rather than on their internal, mostly emotional, experiences^72,73^. People with high scores tend to have a pragmatic, utilitarian approach to life, which is not necessarily totally negative, explaining why this subscale presents mixed results in terms of its relationship to the other two TAS subscales, as well as with the construct of alexithymia itself^74–76^. In the present work, we noticed something interesting with the Externally-oriented subscale. First of all, of all the indicators taken for the external validity of the DERS, this subscale was the one showing the least association with the DERS. This was true regardless of the version we examined (36, 18 or 6 items). The total scores generally correlated but r^2^ for this was barely at the 2% level, in striking contrast with the other observed correlations of the DERS with negative emotionality and symptoms, which could reach 42%. Hence, we think that an externally-oriented thinking could have adaptive aspects by reducing rumination or overemphasis on internal states, potentially aiding in practical problem-solving. For example, individuals with a tendency towards externally-oriented thinking may find it easier to stay focused on tasks and solutions rather than becoming overwhelmed with their emotions. This pragmatic approach can be beneficial in situations that require quick decision-making and action, and where excessive introspection might hinder performance. As a counterpart, however, we noticed also that, for all versions, the difficulty that is mostly associated with the externally-oriented thinking is the lack of awareness subscale (*r* =0.21-0.41, strongly significant in all versions of the DERS). We think that this is a particularly informative result since, indeed, people with externally-oriented thinking often show a lack of introspection and emotional awareness^77^. This suggests a view of externally-oriented thinking that might be nuanced with respect to what was previously discussed. In this view Externally-oriented thinking might contribute to lower emotional awareness (or the other way around) and potentially impede emotional processing and awareness, which are essential for long-term emotional health and effective interpersonal relationships. Without adequate attention to the inner world, individuals may struggle to recognize and address their own emotional needs or those of others, leading to unresolved emotional issues and difficulties in empathy and social interactions. We believe that recognizing the potential duality of externally-oriented thinking is pivotal and future studies could address whether there may be two different concepts (one adaptive and one maladaptive) in the definition of “Externally-oriented thinking”. Balancing these approaches could help individuals harness the strengths of externally-oriented thinking while mitigating its limitations, promoting both practical problem-solving abilities and healthy emotional functioning.

Globally, with the present work, we could successfully provide a valid super short version of the DERS. Our six-item version aligned very well with the other longer-versions of the DERS, both in terms of internal and external validity. Yet, one particular result must be discussed, which is that of the α score, reaching 0.66 in the 6-item version, which is often considered as average. We do not consider this result to be problematic. Indeed, research consistently shows that shorter scales tend to have lower Cronbach’s α values compared to longer scales^78–81^. There are several reasons for this, spurring essentially from what the α exactly measures, and its sensitivity to test length and dimensionality^82,83.^ Indeed, with more items, the impact of any single item variability on the overall scale reliability is diminished. There is hence a balance to be found between shortness of the scale and stability of the measure provided with only one item per dimension, because there are fewer opportunities to average out measurement errors. With the current validation, we have however ensured that the items, though fewer in number, still adequately represent the global construct of emotion regulation difficulties. Conversely, it is well known that Cronbach’s α is influenced by the number of items in a scale. As the number of items increases, α tends to increase, even if the average inter-item correlation remains the same. One needs also to take into account that higher values of Cronbach’s α can also be interpreted as an indication of higher item redundancy^83^, that artificially inflates the α of the entire scale. Again, in our case, the very short version includes only one item per dimension. Hence, by definition, multiple items that redundantly examine one dimension, as in longer versions, will increase the α, and the relative higher independence of the dimensions leads *de facto* to less internal consistency. This calls for a comment on the general score that can be retrieved from the very short form. The total scores we calculated, and this with different algorithms, trying to take into account the different number of items per dimension of the longer version, appeared to be reliably and strongly associated with the total score of the longer versions. However, one can wonder whether it is meaningful to evaluate a total score with this very short form, especially because its total score tends to underestimate the emotion regulation difficulties experienced (see Table 1). We leave it to the researchers to decide, when using the present very short form, whether or not it is appropriate for their research question to use a total score, and, if so, which total score (weighted or unweighted) they choose to calculate. Altogether, having a very short version of the DERS that includes all six dimensions and reliably converges with the longer forms is a significant achievement. This scale offers a practical and efficient tool for quickly assessing emotion regulation difficulties, validated with a large sample of approximately 4000 participants. The ability to provide information on all dimensions in such a concise format is particularly important and pivotal, as it ensures that the scale comprehensively captures the multifaceted nature of the difficulties in emotion regulation. It provides a nuanced understanding of each specific area of difficulty, providing a more complete picture than scales (even longer) that might overlook these aspects. Its brevity and robust validation make it an invaluable resource for researchers and practitioners seeking a quick yet comprehensive measure of emotion regulation, facilitating broader application and accessibility in various research and clinical settings.

This study, while providing valuable insights into the measurement of the difficulties in emotion regulation, has several limitations that should be acknowledged. First, our sample was focused solely on a young adult population aged between 18 and 45 years. This upper age limit was inherited from the inclusion criteria of the original studies, many of which involved physiological recordings. In such studies, individuals over 45 are often excluded to minimize the confounding effects of age-related hormonal and physiological changes. As a result, the generalizability of our findings to broader populations, including older adults and younger children, remains limited. Moreover, since it has been repeatedly shown that levels of anxiety and depression are higher in younger populations^84,85^, relationship with difficulties in emotion regulation may be somewhat different in older age groups. Future research should aim to include a more diverse age range to examine whether the observed difficulties in emotion regulation and their relationship to emotional symptoms, replicate across different developmental stages. Second, our data collection spanned over more than a decade, during which the structure of emotion regulation difficulties may have evolved. Over the course of ten years, numerous external factors, such as advancements in technology, shifts in social media use, changes in educational systems, and major global events (e.g., economic recessions, pandemics), could have had a significant impact on how young people experience and manage their emotions. Furthermore, the cultural context and societal attitudes toward mental health and emotional expression may have shifted during this period, affecting both the reporting and actual experience of emotion regulation difficulties. For instance, increased awareness and reduced stigma surrounding mental health issues could have led to higher reporting of difficulties, while simultaneously fostering better coping mechanisms through improved access to mental health resources. None of these aspects were taken into account here. We believe this is a pivotal question that future studies should tackle, along with considering the temporal dynamics of emotion regulation and investigate how these difficulties may change over time and across different historical contexts. Longitudinal research designs would be particularly valuable in tracking these potential evolutions and understanding the long-term trajectories of emotion regulation skills within individuals. We could also examine different large independent cohorts to identify which emotion regulation difficulties may have changed through the years and in what way. Third, the lack of established measures of emotion regulation difficulties compelled us to use symptomatology as a proxy. There are however other constructs that could have provided additional insights. Specifically, the concept of emotional competence, see for example^86,87^, which encompasses skills such as emotional awareness, expression, and management, could offer an interesting parallel construct to test measurement reliability of emotion regulation difficulties. Future research should endeavor to explore emotional competence as an external validator, as it holds significant potential for enriching our understanding of emotion regulation processes and outcomes.

In conclusion, this study sought to validate the 18-item French version of the DERS as well as to develop and validate a very short version of the DERS (DERS-F SSF), comprising just six items, to provide a quick yet comprehensive measure suitable for contexts with limited time, resources, or participant attention. The validation of the 18-item DERS in French demonstrated robust psychometric properties, aligning closely with its longer counterparts in terms of structural integrity and external validity. Providing the validated DERS-F SSF was equally important. This six-items scale not only aligns well with its longer counterparts in terms of both internal structure and external validity but also retains information on all six dimensions, which is crucial for comprehensively assessing emotion regulation difficulties. Validated with a robust sample size close to 4000 participants, the DERS-F SSF offers researchers and practitioners a practical and efficient tool for assessing emotional dysregulation across diverse contexts. Its brevity ensures quick administration while maintaining sufficient depth to capture the multifaceted nature of emotion regulation challenges. By providing nuanced insights into specific areas of difficulty, this scale enhances our ability to tailor interventions and support strategies effectively. Moving forward, its widespread application holds promise for advancing research and clinical practices aimed at understanding and addressing emotion regulation difficulties in varied populations and settings.

## Methods

### Participants

The data of 3’944 participants were used for this multiple validation study. These come from 21 studies, ran in Swiss French speaking populations. Different sub-samples were used in the present work, depending on the question investigated, which are detailed in the Measures and Data Analyses sections, related to each of the analyses performed. As an overview of the samples analyzed in the present work, Table 8 presents each of the 21 samples, with N and demographic data. Information about other publications pertaining these data for other research questions are mentioned when applicable. Exclusion criteria were generally : pregnancy, medication, and diagnosis of anxiety or mood disorder. Inclusion criteria, which were determined based on the requirement for the different studies, were age between 18 and 45 years old and overall good health. The upper age limit of 45 was applied because most of the original studies involved physiological recordings, and hormonal and physiological changes beyond this age may introduce confounding variability in such measures. The data were gathered from 3007 females (78%) and 827 males (22%), 33 (1%) defined themselves as non-binary or of other genders, the rest of the participants (N=57, 1%) declined to answer or did not provide information for technical reasons. The general goal of this manuscript was to validate a French version of the DERS-SF and to develop a Super Short Form suitable for use across individuals, regardless of gender. As a preliminary step, we nevertheless examined measurement invariance across gender to ensure structural equivalence before proceeding with the validation analyses (see the Data Analyses section below). For all the participants, age averaged 21.3 years. All participants were students, mostly recruited to validate credits in a psychology curriculum. Some participants were compensated with vouchers for their participation.

**Table 8:** Participant demographics for each considered Study.

Study *N*°	Study Code	Total *N*	*N*(%) Female	*N*(%) Male	*N*(%) Other Genders	Mean age (SD)	Recruitment dates	Publication
1	DerED	455	423 (93%)	32 (7%)	--	24.2 (6.1)	01.10.2005–31.12.2007	Dan-Glauser & Scherer^52^
2	AmbS1	71	32 (45%)	33 (46%)	--	21.8 (3.4)	01.03.2016–31.05.2016	Thuillard & Dan-Glauser^88^
3	AmbS2	71	34 (48%)	34 (48%)	--	21.1 (1.7)	01.09.2016–30.11.2016	Thuillard & Dan-Glauser^89^
4	CreaBA	180	136 (76%)	42 (23%)	1 (< 1%)	21.4 (3.0)	01.03.2018–31.03.2018	--
5	AmbS3	69	33 (48%)	32 (46%)	--	21.1 (2.4)	01.03.2017–31.05.2017	Thuillard & Dan-Glauser^90^
6	AmbS4	65	40 (62%)	21 (32%)	--	21.4 (2.1)	01.04.2018–31.05.2018	Thuillard & Dan-Glauser^91^
7	EccA1	500	416 (83%)	80 (16%)	4 (1%)	22.1 (3.2)	01.10.2020–31.05.2021	Sacchi & Dan-Glauser (in press)
8	EccB1	108	80 (74%)	25 (23%)	--	20.8 (2.2)	01.03.2021–31.05.2021	Trentini & Dan-Glauser^92,93^
9	DTMa	134	69 (51%)	12 (9%)	3 (2%)	20.0 (1.7)	01.11.2021–28.02.2022	Sacchi et al. (in prep)
10	EccA2	115	82 (71%)	33 (29%)	--	21.0 (5.5)	01.10.2021–31.01.2022	--
11	EccA3	119	105 (88%)	14 (12%)	--	20.6 (2.5)	01.10.2022–31.12.2022	--
12	EccB2	154	89 (58%)	64 (42%)	--	20.6 (2.7)	01.03.2022–31.05.2023	Trentini & Dan-Glauser^94^
13	EccC1	438	329 (75%)	103 (24%)	3 (1%)	19.3 (1.0)	01.10.2021–30.04.2022	Batziou & Dan-Glauser (in prep)
14	RBMa	166	134 (81%)	29 (17%)	3 (2%)	21.3 (4.9)	01.11.2022–31.01.2023	--
15	EMMa	260	204 (78%)	48 (18%)	8 (3%)	20.5 (3.4)	01.10.2022–31.12.2022	--
16	NMMa	231	194 (84%)	33 (14%)	4 (2%)	20.6 (2.6)	01.11.2022–31.01.2023	--
17	EccB3	103	74 (72%)	29 (28%)	--	22.5 (3.5)	01.03.2023–31.05.2023	Trentini & Dan-Glauser (in prep)
18	EccC2	161	101 (63%)	60 (37%)	--	21.2 (3.3)	01.12.2022–30.04.2023	--
19	OcdMa	248	209 (84%)	37 (15%)	2 (1%)	20.4 (3.1)	01.10.2020–31.12.2020	Salamon, et al^95^.
20	MSMa	240	201 (84%)	32 (13%)	5 (2%)	20.7 (3.2)	01.11.2023–31.12.2023	--
21	EccE2	56	22 (39%)	34 (61%)	--	23.1 (2.6)	01.09.2023–31.01.2024	--

Discrepancies between Total N and split per gender are due to participants declining to indicate gender or for whom we were not able to collect the information.

### Measures

#### Difficulties in emotion regulation

##### Difficulty in emotion regulation scale 36 items (DERS-36)

Both the original English version^18^ and the translated French version of the DERS^52^ have 36 items. The instrument estimates the severity of the deficits in emotion regulation domains. All items are scored on a five-point Likert scale from 1 = almost never to 5 = almost always, which indicates the frequency of the behavior described in each item. The instrument assesses six underlying dimensions, which have been described in detail in the introductory section: (1) Nonacceptance of emotional response (indicated hereafter as Non-acceptance), (2) Difficulties in adopting goal-directed behaviors (indicated hereafter as Goals), (3) Difficulties in controlling impulsive behaviors (indicated hereafter as Impulse), (4) Lack of emotional awareness (indicated hereafter as Awareness), (5) Limited access to emotion regulation strategies (indicated hereafter as Strategies), and (6) Lack of clarity (indicated hereafter as Clarity). Internal consistencies (Cronbach’s α) have been found to be quite high (0.93 in the original version, 0.92 in the translated one). Each factor of the two scales had an internal consistency greater than 0.74. Using intra-class correlation coefficients, a test-retest reliability analysis with 21 participants and a 4- to 8-week interval between tests (English), or with 41 participants and 9-week interval between tests (French) revealed a strong reproducibility of the scores (ρ = 0.88 for both validations). For the present report, studies N° 1–6, 8, and 12–21 (*N* = 3076, 2333 females, 76%, 710 men, 23%, mean age = 21.2) all included the French DERS 36-item version^52^.

##### Difficulty in Emotion Regulation Scale 18 items (DERS-SF).

As reported in the introduction, Kaufman and colleagues ^42^proposed a short version of the DERS with 18 items, called DERS-SF (not to be confounded with the DERS-18, which is another version). The DERS-SF has a high internal consistency (α=0.89-0.91) and had rather high correlations with the original instruments (0.90-0.98). For the present work, we had two different kinds of data regarding the DERS-SF that we wanted to validate in French. First, for studies N° 7 and 9-11 (N=868, 672 females, 77%, 139 men, 16%, mean age = 21.4) we had to administer the French DERS-SF to the participants, even though it had not yet been validated, due to time constraints. For this, we selected the corresponding 18 items suggested by Kaufman and colleagues^42^, but using the French formulation of these items. Inclusion of these data in the present work permitted to observe the standalone scores of the DERS-SF (not filled together with the other 18 items of the 36 item version), its structure, and the way its scores are associated with other questionnaires. We called this *Standalone F-DERS-SF*. For all the other studies N° (1–6, 8, and 12–21), we selected, from the DERS-36 that we participants were asked to fill, the scores of the 18 items corresponding to Kaufman’s 18-item version. This allowed the validation of the French translation of the DERS-SF with many more participants (+ *N* = 3076). Yet, one important point needs to be stressed. It is not possible to ensure that the 18 items, filled among 36, are filled exactly the same way as they would if they were presented as a standalone 18-item version. It is however considered here as a reasonable assumption. We called this subset the *Extracted F-DERS-SF*. We also conducted the analyses on both subsets, called the *Full Sample.*

#### External validity measures

To assess the external validity of the emotion regulation difficulty scale, we examined its associations with affect, two of the most prevalent emotional symptoms (depression and anxiety), and alexithymia. Indeed, higher negative affect and lower positive affect have been shown to be linked to greater emotion regulation challenges^96,97^. Similarly, since emotion regulation deficits are central to anxiety and depression^98–100^, strong associations with these constructs would further support the scale’s validity. Finally, since alexithymia, characterized by difficulties in identifying and expressing emotions, is intricated with some emotion regulation difficulties^101–103^, we deemed this measurement appropriate for an external validity check.

##### Positive and negative affect schedule (PANAS)

In addition to the DERS, participants in studies N° 2–6, 8, 10–14, 17–18 and 21 (*N*= 1876, 1289 females, 69%, 575 males, 31%, mean age = 20.8) also completed the Positive and Negative Affect Scale PANAS^104^. The PANAS proposes 20 affects or feelings (10 negative and 10 positive), such as afraid, upset, enthusiastic or proud for which participants needed to rate to what extent they feel in such way from 1 (Not at all) to 5 (Extremely). Participant can receive the instruction to consider a specific timeframe for reporting the frequency of these affects (today, past two weeks, etc.). In the present work, only the general affect frequency was considered (“Indicate how often you feel the proposed emotions *in general*”). From the 20 items, two scores are created, representing the general frequency of negative affect, and the general frequency of positive affect.

##### Anxiety and depression levels (PHQ-4)

For participants included in studies N° 8–14, 16–18 and 20 (*N*= 1969, 1456 females, 74%, 456 males, 23%, mean age = 20.5), we measured their Anxiety and Depression level with the 4-item Patient Health Questionnaire PHQ-4^105^. The PHQ-4 is a condensed version of the PHQ-9 for depression^106^, and the GAD-7 for anxiety^107^, and assesses symptoms of Anxiety with 2 items and symptoms of Depression with 2 items on a scale from 0 to 3, evaluating the frequency with which participants felt worry, anxiety or tension (anxiety), sadness, despair and no interest/pleasure in doing things (depression) in the last two weeks. Two scores, Depression and Anxiety, are considered for this present work.

##### Toronto alexithymia scale (TAS-20)

In studies N° 2, 3, 5, 6, 8–14, 16–18 and 20 (*N* = 2245, 1595 females, 71%, 576 males, 26%, mean age = 20.6) we also obtained from participants their self-reported alexithymia level, with the French version^108^ of the Toronto Alexithymia Scale TAS-20^73^. The TAS-20 is designed to assess alexithymia, i.e., the difficulty in identifying and describing emotions. It consists of 20 items describing a difficulty in alexithymia domains such as “I am often confused about what emotion I am feeling” or “I am often puzzled by sensations in my body”. Participants are required to score on a 5-point Likert scale (1–5) to what extent they agree with the proposition. The TAS-20 includes three subscales, each focusing on a different aspect of alexithymia: (a) Difficulty Identifying Feelings, (b) Difficulty Describing Feelings (generally to others), and (c) Externally-Oriented Thinking, which evaluates the tendency to focus attention externally rather than internally on one’s own thoughts and feelings.

### Procedure

Except for study N° 1 (where questionnaires were presented on paper), all questionnaires were completed online. All participants were informed about the details of the procedure and of their task, risk, benefits and right to withdraw prior completing the survey. All participants included here gave their informed consent, including for data re-use, and did not revoke it afterwards. After signing (on paper or electronically) the consent form, questionnaires were presented in randomized order. Participants had to follow the instructions and give responses by clicking on their desired answer for all items. At the end of the surveys, all participants were thanked and debriefed about the purpose of the respective studies and could enlist on a separate list if they wished to receive the results of the study once processed. Except for Study 1, which was conducted before the establishment of local ethics committees, all study protocols were approved by the appropriate institutional or regional ethics committees: approval number CER-VD 2015-00071 (*Commission Cantonale d’Ethique de la recherche sur l’Etre Humain du canton de Vaud*), and approval numbers C-SSP-042020-00001, C_SSP_112020_00006, C_SSP_032021_00009, C_SSP_082021_00004, C-SSP_062022_00008 (all from the *Commission d’Ethique de la Recherche de la Faculté des Sciences Sociales et Politiques de l’Université de Lausanne*). Some approvals applied to multiple studies. All data were gathered in accordance with National guidelines/regulations and have been performed in accordance with the Declaration of Helsinki.

### Data analyses

All analyses were performed with Jamovi version 2.4.8 and 2.7.6^109^. All data were first checked for outliers and normality distributions. The analyses and results focused on two different aspects : (a) the data of the DERS-SF, for its validation in French language (testing of H1 and H2) and resulting in the F-DERS-SF, and (b) the examination of the DERS with 36 items to elaborate a Super-Short-Form French DERS (DERS-F SSF), created here. Through this latter analysis we aimed to test H3 and H4, i.e., validating the six items representing the most the original structure and subscales. The approach for validating the DERS-SF in French is similar to the ones used for the 36-item versions (both in English and French), and for the short form in English. To ease scores comparisons, all subscales and total scores issued from the DERS (any form) are calculated as averages, hence ranging on a scale from 1 to 5.

Prior to testing the main hypothesis, all descriptives have been calculated. Then, measurement invariance across gender was tested sequentially (configural, metric, scalar) using multi-group confirmatory factor analyses. Invariance was evaluated based on changes in model fit indices, with thresholds of ΔCFI ≤ 0.010^110^ and ΔRMSEA ≤ 0.015, complemented by ΔSRMR ≤ 0.030 for metric and ≤ 0.015 for scalar invariance^111^.

In order to test H1, we took advantage of both exploratory and confirmatory factor analyses. We first conducted exploratory factor analyses (EFA), using Maximum Likelihood estimation, with Varimax rotation, without constraining for factor numbers (leaving it up to parallel analyses). We then conducted confirmatory factor analysis (CFA) to evaluate the quality of the model imposed by the original SF versions. Despite conducting EFA and CFA on same dataset is not common, this approach is particularly useful when adapting an existing scale as it investigates both if the translated version would offer a different structure from the original and whether original theoretical dimensions hold empirically, see^112^ for more information. These two analyses were performed (1) on the *Full Sample* (*N* = 3944), then (2) on the *Standalone F-DERS-SF* (*N* = 868), and then (3) on the *Extracted F-DERS-SF* (*N* = 3076, see the Measures section for explanation about these terminologies).

As a second step of testing H1, we evaluated the internal reliability by computing the Cronbach α of the scale and each of the 6 subscales. At this stage, we also delved into the effect of the potential removal of problematic items on alphas, such items identified by the non-constrained EFA. Again, all analyses were performed (1) on the *Full Sample*, (2) on the *Standalone F-DERS-SF*, and (3) on the *Extracted F-DERS-SF*.

Following the same rationale, the mean inter-item correlation for the full scale and each subscale was also computed, as well as the correlation between (a) each item and the total score and (b) each item and the score of the subscale it belongs to. For this step, a corrected correlation was provided, in the sense that the correlation of the item was computed with the total or subscale scores obtained after the removal of the considered item. Again, all analyses were performed (1) on the *Full Sample*, (2) on the *Standalone F-DERS-SF*, and (3) on the *Extracted F-DERS-SF*.

A final step of the testing of H1 was to come back to the 36-item version and to calculate Tucker’s ϕ (congruence index) for the two matrices of loading (the DERS-F 36 items, and the F-DERS-SF), obtained on the constrained EFA (6 factors). Of course, only the common 18 items are considered. These analyses allowed to observe if factor sub-structure is similar depending on whether items are taken from a 36- or an 18-item version. Three Tucker’s ϕ were computed between the DERS-F 36 items and (1) the *Full Sample*, (2) the *Standalone F-DERS-SF*, and (3) the *Extracted F-DERS-SF*.

In order to test H2 (external validity of the F-DERS-SF) we computed Pearson’s correlation coefficients examining the relationships between the DERS-F scores (total and subscales) and the corresponding *Extracted F-DERS-SF* scores. Then, Pearson’s correlations were computed to analyze the relationships between the total and subscale scores of (1) the *Full Sample*, (2) the *Standalone F-DERS-SF*, and (3) the *Extracted F-DERS-SF* on the one hand, and the scores provided by the PANAS (positive and negative affects), the PHQ-4 (anxiety and depression) and the TAS-20 (alexithymia) on the other hand.

For the second goal of the study, i.e., the creation of a 6 items DERS-F super short form, we first focused on the studies for which participants filled the full 36-item version. The six items were therefore extracted only from Studies N° 1–6, 8, and 12–21 (*N* = 3076). We first conducted exploratory factor analyses (EFA) using principal axis factoring with Promax rotation on the 36-item data, but with the constraint of obtaining six factors. After having checked that the six factors corresponded to the validated underlying structure, the maximally loaded items of each subscale were retained for the super-short form. After this decision, the six corresponding item scores in our studies were extracted and composed the *Extracted DERS-F SSF*. Each item corresponded to a subscale. For the calculation of the total score of the *DERS-F SSF*, two variants were tested to see which total score that was most comparable to other forms. Indeed, we imagined that researchers may like, at some point, to compare their results obtained with the *DERS-F SSF* with results of colleagues that used other DERS forms. Hence, to be able to refer to past results using different instruments, we also tried to obtain a more comparable total score. The first Total-Score (TS_Unweighted) is simply an average of the six-item scores. Since the original scale has an imbalanced number of items per subscale (for example, eight items for Strategies but five for Non-Acceptance), the total score of the scale gains more weight from items belonging to certain subscales. To counterbalance this, we calculated another total score (TS_Weighted) to see whether it would bring more validity. TS_Weighted takes into account the six subscales but giving a weight of 6 to Non acceptance, Impulse and Awareness, a weight of 5 for Goals and Clarity, and a weight of 8 for Strategies. From the extracted 6 subscale scores, and assuming that these would constitute participants’ responses to a scale that would only contain six items, we tested H3 by evaluating the general Cronbach α of such scale. Mean inter-item correlation and corrected item-total score correlation for each item (corrected as explained above, and with each time TS weighted and unweighted taken into account) were also computed.

As a last step, Pearson’s correlations between the 36-item version subscale scores and their corresponding 6-item version scores, as well as between the total scores of both scales, were computed. For this very last step, and in order to compare all versions together, we computed all the correlations between the *DERS-F SSF* and the *DERS-F* 36 items, as well as the 18-item version for the full sample.

Finally, to test H4, the new *DERS-F SSF* scores were correlated (Pearson’s r) with the scores provided by the PANAS (positive and negative affects), the PHQ-4 (anxiety and depression) and the TAS-20 (alexithymia). These correlations were also compared to the ones found for testing H2, observing the difference in external validity between the present *DERS-F SSF* and its longer versions.

## Supplementary Information

Below is the link to the electronic supplementary material.

Supplementary Material 1

## Data Availability

The data and materials are available in the OSF repository [https://osf.io/zsurw/](https:/osf.io/zsurw).

## References

[1] Russell, J. A. Core affect and the psychological construction of emotion. *Psychol. Rev.* **110**, 145–172. 10.1037/0033-295x.110.1.145 (2003). 12529060 10.1037/0033-295x.110.1.145

[2] Kappas, A. Emotion and regulation are One! *Emot. Rev.* **3**, 17–25 (2011).

[3] Gross, J. J. The emerging field of emotion regulation: an integrative review. *Rev. Gen. Psychol.* **2**, 271–299. 10.1037/1089-2680.2.3.271 (1998).

[4] Gratz, K. L. & Tull, M. T. A clinically useful conceptualization of emotion regulation grounded in functional contextualism and evolutionary theory. *World Psychiatry*. **21**, 460–461. 10.1002/wps.21021 (2022). 36073681 10.1002/wps.21021 PMC9453920

[5] Troy, A. S., Shallcross, A. J. & Mauss, I. B. A person-by-situation approach to emotion regulation. *Psychol. Sci.* **24**, 2505–2514 (2013). 24145331 10.1177/0956797613496434

[6] Yunus, M. & Chaudhary, P. The role of emotion regulation in stress management: an overview. *J. Clin. Res. Appl. Med.* **3**, 9–12. 10.5530/jcram.3.1.3 (2023).

[7] Donahue, J. J., McClure, K. S. & Moon, S. M. The relationship between emotion regulation difficulties and psychopathic personality characteristics. *Personal. Disord*. **52**, 186–194 (2014). 10.1037/per0000025 24341861

[8] Igra, L. et al. Examining the associations between difficulties in emotion regulation and symptomatic outcome measures among individuals with different mental disorders. *Front. Psychol.* **14**. 10.3389/fpsyg.2023.944457 (2023). 10.3389/fpsyg.2023.944457 PMC10043222 36998365

[9] Sheppes, G., Suri, G. & Gross, J. J. Emotion regulation and psychopathology. *Ann. Rev. Clin. Psychol.* **11**, 379–405 (2015). 25581242 10.1146/annurev-clinpsy-032814-112739

[10] Tull, M. T., Barrett, H. M., McMillan, E. S. & Roemer, L. A preliminary investigation of the relationship between emotion regulation difficulties and posttraumatic stress symptoms. *Behav. Ther.* **38**, 303–313 (2007). 17697854 10.1016/j.beth.2006.10.001

[11] Aldao, A., Nolen-Hoeksema, S. & Schweizer, S. Emotion-regulation strategies across psychopathology: A meta-analytic review. *Clin. Psychol. Rev.* **30**, 217–237. 10.1016/j.cpr.2009.11.004 (2010). 20015584 10.1016/j.cpr.2009.11.004

[12] Aldao, A. & Nolen-Hoeksema, S. When are adaptive strategies most predictive of psychopathology? *J. Abnorm. Psychol.* **121**, 276–281. 10.1037/a0023598 (2012). 21553934 10.1037/a0023598

[13] Cisler, J. M. & Olatunji, B. O. Emotion regulation and anxiety disorders. *Curr. Psychiatry Rep.* **14**, 182–187 (2012). 22392595 10.1007/s11920-012-0262-2 PMC3596813

[14] Garke, M. Å. et al. Emotion dysregulation across levels of substance use. *Psychiatry Res.* **296**. 10.1016/j.psychres.2020.113662 (2020). 10.1016/j.psychres.2020.113662 33406445

[15] Sloan, E. et al. Emotion regulation as a transdiagnostic treatment construct across anxiety, depression, substance, eating and borderline personality disorders: A systematic review. *Clin. Psychol. Rev.* **57**, 141–163 (2017). 28941927 10.1016/j.cpr.2017.09.002

[16] Heleniak, C., Jenness, J. L., Vander Stoep, A., McCauley, E. A. & McLaughlin, K. A. Childhood maltreatment exposure and disruptions in emotion regulation: A transdiagnostic pathway to adolescent internalizing and externalizing psychopathology. *Cogn. Therapy Res.* **40**, 394–415 (2016). 10.1007/s10608-015-9735-z PMC5042349 27695145

[17] Miu, A. C. et al. Emotion regulation as mediator between childhood adversity and psychopathology: A meta-analysis. *Clin. Psychol. Rev.* **93** (2022). 10.1016/j.cpr.2022.102141 PMC8960368 35219929

[18] Gratz, K. L. & Roemer, L. Multidimensional assessment of emotion regulation and dysregulation: Development, factor structure, and initial validation of the difficulties in emotion regulation scale. *J. Psychopathol. Behav. Assess.* **26**, 41–54. 10.1023/b:Joba.0000007455.08539.94 (2004).

[19] Lopes, P. N., Salovey, P., Côté, S. & Beers, M. Emotion regulation abilities and the quality of social interaction. *Emotion* **5**, 113–118 (2005). 15755224 10.1037/1528-3542.5.1.113

[20] Visted, E. et al. The association between self-reported difficulties in emotion regulation and heart rate variability: the salient role of not accepting negative emotions. *Front. Psychol.* **8**. 10.3389/fpsyg.2017.00328 (2017). 10.3389/fpsyg.2017.00328 PMC5343522 28337160

[21] Subic-Wrana, C. et al. How is emotional awareness related to emotion regulation strategies and self-reported negative affect in the general population? *PLoS One*. **9**. 10.1371/journal.pone.0091846 (2014). 10.1371/journal.pone.0091846 PMC3956759 24637792

[22] Vine, V. & Aldao, A. Impaired emotional clarity and psychopathology: A transdiagnostic deficit with symptom-specific pathways through emotion regulation. *J. Soc. Clin. Psychol.* **33**, 319–342 (2014).

[23] Amy, L. B., Ruby, J. B. & Maree, J. A. Overcoming difficulties in measuring emotional regulation: assessing and comparing the psychometric properties of the DERS long and short forms. *Cogent Psychol.* **9** (2022).

[24] Fowler, J. et al. Construct validity and factor structure of the difficulties in emotion regulation scale among adults with severe mental illness. *J. Psychiatr. Res.* **58**, 175–180. 10.1016/j.jpsychires.2014.07.029 (2014). 25171941 10.1016/j.jpsychires.2014.07.029

[25] Neumann, A., van Lier, P. A., Gratz, K. L. & Koot, H. M. Multidimensional assessment of emotion regulation difficulties in adolescents using the difficulties in emotion regulation scale. *Assessment* **17**, 138–149 (2010). 19915198 10.1177/1073191109349579

[26] Osborne, T. L., Michonski, J., Sayrs, J., Welch, S. S. & Anderson, L. K. Factor structure of the difficulties in emotion regulation scale (DERS) in adult outpatients receiving dialectical behavior therapy (DBT). *J. Psychopathol. Behav. Assess.* **39**, 355–371 (2017).

[27] Mekawi, Y. et al. Validation of the difficulties with emotion regulation scale in a sample of trauma-exposed black women. *J. Clin. Psychol.* **77**, 587–606 (2021). 32762085 10.1002/jclp.23036

[28] Bardeen, J. R., Fergus, T. A. & Orcutt, H. K. An examination of the latent structure of the difficulties in emotion regulation scale. *J. Psychopathol. Behav. Assess.* **34**, 382–392. 10.1007/s10862-012-9280-y (2012).

[29] Lee, D. J., Witte, T. K., Bardeen, J. R., Davis, M. T. & Weathers, F. W. A factor analytic evaluation of the difficulties in emotion regulation scale. *J. Clin. Psychol.* **72**, 933–946. 10.1002/jclp.22297 (2016). 27018649 10.1002/jclp.22297

[30] Ritschel, L. A., Tone, E. B., Schoemann, A. M. & Lim, N. E. Psychometric properties of the difficulties in emotion regulation scale across demographic groups. *Psychol. Assess.* **27**, 944–954. 10.1037/pas0000099 (2015). 25774638 10.1037/pas0000099

[31] Sörman, K. et al. Measures of emotion regulation: convergence and psychometric properties of the difficulties in emotion regulation scale and emotion regulation questionnaire. *J. Clin. Psychol.* **78**, 201–217. 10.1002/jclp.23206 (2022). 34217149 10.1002/jclp.23206

[32] Pierce, T., Elmallah, R., Cherian, J., Jauregui, J. & Michael, A. M. Standardized questionnaire time burden for practitioners and patients. *Surg. Technol. Int.* **26**, 302–306 (2015). 26055024

[33] Herzog, A. R. & Bachman, J. Effects of questionnaire length on response quality. *Pub. Opin. Q.* **45**, 549–559 (1981).

[34] Rolstad, S., John, A. & Rydén, A. Response burden and questionnaire length: is shorter better? A review and meta-analysis. *Value Health*. **14**, 1101–1108 (2011). 22152180 10.1016/j.jval.2011.06.003

[35] Galesic, M. & Bošnjak, M. Effects of questionnaire length on participation and indicators of response quality in a web survey. *Pub. Opin. Q.* **73**, 349–360 (2009).

[36] Victor, S. E. & Klonsky, E. D. Validation of a brief version of the difficulties in emotion regulation scale (DERS-18) in five samples. *J. Psychopathol. Behav. Assess.* **38**, 582–589 (2016). 10.1007/s10862-015-9514-x PMC4882111 27239096

[37] Bjureberg, J. et al. Development and validation of a brief version of the difficulties in emotion regulation scale: the DERS-16. *J. Psychopathol. Behav. Assess.* **38**, 284–296. 10.1007/s10862-015-9514-x (2016). 27239096 10.1007/s10862-015-9514-x PMC4882111

[38] Burton, A. L., Brown, R. & Abbott, M. J. Overcoming difficulties in measuring emotional regulation: assessing and comparing the psychometric properties of the DERS long and short forms. *Cogent Psychol.* **9** (2022).

[39] Hallion, L. S., Steinman, S. A., Tolin, D. F. & Diefenbach, G. J. Psychometric properties of the difficulties in emotion regulation scale (DERS) and its short forms in adults with emotional disorders. *Front. Psychol.* **9**. 10.3389/fpsyg.2018.00539 (2018). 10.3389/fpsyg.2018.00539 PMC5917244 29725312

[40] Raimondi, G. et al. Meta-analysis of the difficulties in emotion regulation scale and its short forms: A two-part study. *Journal Clin. Psychology Adv. Online Publication*. **24**. 10.1002/jclp.23695 (2024). 10.1002/jclp.23695 38630901

[41] Skutch, J. M., Wang, S. B., Buqo, T., Haynos, A. F. & Papa, A. Which brief is best? Clarifying the use of three brief versions of the difficulties in emotion regulation scale. *J. Psychopathol. Behav. Assess.* **41**, 485–494. 10.1007/s10862-019-09736-z (2019). 34446987 10.1007/s10862-019-09736-z PMC8386519

[42] Kaufman, E. A. et al. The difficulties in emotion regulation scale short form (DERS-SF): validation and replication in adolescent and adult samples. *J. Psychopathol. Behav. Assess.* **38**, 443–455. 10.1007/s10862-015-9529-3 (2016). 10.1007/s10862-015-9529-3 PMC12788809 41522882

[43] Asgarizadeh, A., Mazidi, M., Preece, D. A. & Dehghani, M. Construct validity and measurement invariance of the difficulties in emotion regulation Scale - Short form (DERS-SF): further evidence from community and student samples. *J. Personal. Assess. Adv. Online Publ*. **11**. 10.1080/00223891.2024.2340506 (2024). 10.1080/00223891.2024.2340506 38647207

[44] Gouveia, P., Ramos, C., Brito, J., Almeida, T. C. & Cardoso, J. The difficulties in emotion regulation Scale – Short form (DERS-SF): psychometric properties and invariance between genders. *Psicol.: Reflex. Crít.*. **35**. 10.1186/s41155-022-00214-2 (2022). 10.1186/s41155-022-00214-2 PMC9076757 35522349

[45] Moreira, H., Gouveia, M. J. & Canavarro, M. C. A bifactor analysis of the difficulties in emotion regulation Scale-Short form (DERS-SF) in a sample of adolescents and adults. *Curr. Psychol.* **41**, 757–782. 10.1007/s12144-019-00602-5 (2022).

[46] Matsumoto, D. in *In the Handbook of Culture and Psychology*. 171–194 (eds Matsumoto, D.) (Oxford University Press, 2001).

[47] Rosharudin, N. A. et al. Psychometric properties of the Malay version of the difficulties in emotion regulation Scale-18 in Malaysian adolescents. *PLoS One*. **18**, e0289551. 10.1371/journal.pone.0289551 (2023). 37639447 10.1371/journal.pone.0289551 PMC10461853

[48] Fekih-Romdhane, F., Kanj, G., Obeid, S. & Hallit, S. Psychometric properties of an Arabic translation of the brief version of the difficulty in emotion regulation scale (DERS-16). *BMC Psychol.* **11**. 10.1186/s40359-023-01117-2 (2023). 10.1186/s40359-023-01117-2 PMC10015724 36922893

[49] Reivan-Ortiz, G. G. & Rodas, O. Reivan Ortiz, P. N. A brief version of the difficulties in emotion regulation scale (DERS): validity evidence in Ecuadorian population. *Int. J. Psychol. Res.* **13**, 14–24. 10.21500/20112084.4325 (2020). 10.21500/20112084.4325 PMC7735515 33329874

[50] Giromini, L., Velotti, P., de Campora, G., Bonalume, L. & Cesare Zavattini, G. Cultural adaptation of the difficulties in emotion regulation scale: reliability and validity of an Italian version. *J. Clin. Psychol.* **68**, 989–1007. 10.1002/jclp.21876 (2012). 22653763 10.1002/jclp.21876

[51] Mazaheri, M. Psychometric properties of the Persian version of the difficulties in emotion regulation scale (DERS-6 & DERS-5- Revised) in an Iranian clinical sample. *Iran. J. Psychiatry*. **10**, 115–122 (2015). 26884788 PMC4752524

[52] Dan-Glauser, E. S. & Scherer, K. R. The difficulties in emotion regulation scale (DERS): factor structure and consistency of a French translation. *Swiss J. Psychol.* **72**, 5–11. 10.1024/1421-0185/a000093 (2013).

[53] Côté, G., Gosselin, P. & Dagenais, I. Évaluation multidimensionnelle de La régulation des émotions: propriétés psychométriques d’une version Francophone du difficulties in emotion regulation scale. *J. De Thérapie Comportementale Et Cogn.* **23**, 63–72. 10.1016/j.jtcc.2013.01.005 (2013).

[54] Park, H. & Hill, R. B. Development and validation of a short form of the occupational work ethic inventory. *J. Career Tech. Educ.* **32**, 9–28 (2018).

[55] Rammstedt, B. & Beierlein, C. Can’t we make it any shorter? *J. Individual Differences*. **35**, 212–220 (2014).

[56] Koğar, H. Development of a short form: Methods, examinations, and recommendations. *J. Meas. Evaluation Educ. Psychol.* **11**, 302–310. 10.21031/epod.739548 (2020).

[57] Bowling, A. Just one question: if one question works, why ask several? Single compared with multi-item measures. *J. Epidemiol. Commun. Health*. **59**, 342–345. 10.1136/jech.2004.021204 (2005). 10.1136/jech.2004.021204 PMC1733095 15831678

[58] Boynton, P. M. & Greenhalgh, T. Selecting, designing, and developing your questionnaire. *BMJ: Br. Med. J.* **328**, 1312–1315 (2004). 15166072 10.1136/bmj.328.7451.1312 PMC420179

[59] Vold, J. H. et al. Validation of a three-item fatigue severity scale for patients with substance use disorder: a cohort study from Norway for the period 2016–2020. *Health Qual. Life Outcomes* **19** (2020). 10.1186/s12955-021-01708-w PMC7923309 33653349

[60] Espirito-Santo, H. et al. Emotion dysregulation in older people: validity and reliability of an 8-item version of the difficulties in emotion regulation scale. *Aging Ment. Health*. **28**, 360–368. 10.1080/13607863.2023.2260329 (2024). 37771115 10.1080/13607863.2023.2260329

[61] Penner, F., Steinberg, L. & Sharp, C. The development and validation of the difficulties in emotion regulation Scale-8: providing respondents with a uniform context that elicits thinking about situations requiring emotion regulation. *J. Pers. Assess.* **105**, 657–666. 10.1080/00223891.2022.2133722 (2023). 36306434 10.1080/00223891.2022.2133722

[62] Zimmerman, M. et al. Developing brief scales for use in clinical practice: the reliability and validity of single-item self-report measures of depression symptom severity, psychosocial impairment due to depression, and quality of life. *J. Clin. Psychiatry*. **67**, 1536–1541. 10.4088/jcp.v67n1007 (2006). 17107244 10.4088/jcp.v67n1007

[63] Bertolotti, G. et al. Shortened questionnaires to assess anxiety and depression during in-hospital rehabilitation: clinical validation and cutoff scores. *Neuropsychiatr. Dis. Treat.* **12**, 2627–2633 (2016). 27789951 10.2147/NDT.S111797 PMC5068471

[64] Bardeen, J. R., Fergus, T. A., Hannan, S. M. & Orcutt, H. K. Addressing psychometric limitations of the difficulties in emotion regulation scale through item modification. *J. Pers. Assess.* **98**, 298–309 (2016). 26538407 10.1080/00223891.2015.1091774

[65] Cho, Y. & Hong, S. The new factor structure of the Korean version of the difficulties in emotion regulation scale (K-DERS) incorporating method factor. *Meas. Evaluation Couns. Dev.* **46**, 192–201 (2013).

[66] Medrano, L. A. & Trógolo, M. Construct validity of the difficulties in emotion regulation scale: further evidence using confirmatory factor analytic approach. *Abnormal Behav. Psychol.* **2** (2016).

[67] Monell, E., Birgegård, A., Nordgren, L., Hesser, H. & Bjureberg, J. Factor structure and clinical correlates of the original and 16-item version of the difficulties in emotion regulation scale in adolescent girls with eating disorders. *J. Clin. Psychol.* **78**, 1201–1219. 10.1002/jclp.23286 (2022). 34855219 10.1002/jclp.23286

[68] Li, J., Han, Z. R., Gao, M. M., Sun, X. & Ahemaitijiang, N. Psychometric properties of the Chinese version of the difficulties in emotion regulation scale (DERS): factor structure, reliability, and validity. *Psychol. Assess.* **30**, e1–e9. 10.1037/pas0000582 (2018). 29756796 10.1037/pas0000582

[69] Ruganci, R. N. & Gençöz, T. Psychometric properties of a Turkish version of the difficulties in emotion regulation scale. *J. Clin. Psychol.* **66**, 442–455. 10.1002/jclp.20665 (2010). 20143312 10.1002/jclp.20665

[70] Danasasmita, F. S. et al. Validity and reliability of the difficulties in emotion regulation scale short form in Indonesian non-clinical population. *Front. Psychiatry* **15** (2024). 10.3389/fpsyt.2024.1380354 PMC11000630 38590788

[71] Eckland, N. S. & Thompson, R. J. State emotional clarity is an indicator of fluid emotional intelligence ability. *J. Intell.* **11**. 10.3390/jintelligence11100196 (2023). 10.3390/jintelligence11100196 PMC10607947 37888428

[72] Kekkonen, V. et al. Externally oriented thinking style increases primary health care use in adolescence. *Eur. J. Pub. Health*. **33**, 418–423. 10.1093/eurpub/ckad041 (2023). 36977353 10.1093/eurpub/ckad041 PMC10234664

[73] Bagby, R. M., Parker, J. D. A. & Taylor, G. J. The Twenty-Item Toronto alexithymia Scale - I. Item selection and cross-validation of the factor structure. *J. Psychosom. Res.* **38**, 23–32 (1994). 8126686 10.1016/0022-3999(94)90005-1

[74] Gignac, G. E., Palmer, B. R. & Stough, C. A confirmatory factor analytic investigation of the TAS–20: corroboration of a five-factor model and suggestions for improvement. *J. Pers. Assess.* **89**, 247–257 (2007). 18001225 10.1080/00223890701629730

[75] Preece, D. A., Becerra, R., Robinson, K. & Dandy, J. Assessing alexithymia: psychometric properties and factorial invariance of the 20-Item Toronto alexithymia scale in nonclinical and psychiatric samples. *J. Psychopathol. Behav. Assess.* **40**, 276–287 (2017).

[76] Meganck, R., Vanheule, S., Desmet, M. & Inslegers, R. Does the 20-Item Toronto alexithymia scale measure alexithymia? A study of natural Language use. *Psychol. Rep.* **105**, 945–956 (2009). 20099558 10.2466/PR0.105.3.945-956

[77] Trentini, C., Tambelli, R., Maiorani, S. & Lauriola, M. Gender differences in empathy during adolescence: does emotional self-awareness matter? *Psychol. Rep.* **125**, 913–936. 10.1177/0033294120976631 (2021). 33588646 10.1177/0033294120976631

[78] Bakker, B. N. & Lelkes, Y. Selling ourselves short? How abbreviated measures of personality change the way we think about personality and politics. *J. Politics*. **80**, 1311–1325. 10.1086/698928 (2018).

[79] Franke, G. R., Rapp, A. & Andzulis, J. M. Using shortened scales in sales research: Risks, benefits, and strategies. *J. Personal Sell. Sales Manage.* **33**, 319–328. 10.2753/pss0885-3134330306 (2013).

[80] Xiao, L., Hau, K. T. & Wang, M. D. Revisiting the usage of alpha in scale evaluation: effects of scale length and sample size. *Educ. Meas.* **43**, 74–81. 10.1111/emip.12604 (2024).

[81] Ziegler, M., Poropat, A. & Mell, J. Does the length of a questionnaire matter? *J. Individ. Diff.*. **35**, 250–261. 10.1027/1614-0001/a000147 (2014).

[82] Streiner, D. L. Starting at the beginning: an introduction to coefficient alpha and internal consistency. *J. Pers. Assess.* **80**, 99–103. 10.1207/s15327752jpa8001_18 (2013). 10.1207/S15327752JPA8001_18 12584072

[83] Tavakol, M. & Dennick, R. Making sense of cronbach’s alpha. *Int. J. Med. Educ.* **2**, 53–55. 10.5116/ijme.4dfb.8dfd (2011). 28029643 10.5116/ijme.4dfb.8dfd PMC4205511

[84] Christensen, H. et al. Age differences in depression and anxiety symptoms: a structural equation modelling analysis of data from a general population sample. *Psychol. Med.* **29**, 325–339 (1999). 10218924 10.1017/s0033291798008150

[85] Wuthrich, V. M., Johnco, C. J. & Wetherell, J. L. Differences in anxiety and depression symptoms: comparison between older and younger clinical samples. *Int. Psychogeriatr.* **27**, 1523–1532 (2015). 25892278 10.1017/S1041610215000526

[86] Davis, S. K. & Qualter, P. in *In the Encyclopedia of Child and Adolescent Development*. (eds Hupp, S. & Jewell, J. D.) (Wiley, 2020).

[87] Verbeke, W., Belschak, F. & Bagozzi, R. P. Exploring emotional competence: its effects on coping, social capital, and performance of sales people. *Rep. Ser. Res. Manag.* **20** (2004).

[88] Thuillard, S. & Dan-Glauser, E. The regulatory effect of choice in situation selection reduces experiential, exocrine and respiratory arousal for negative emotional stimulations. *Sci. Rep.* **7**10.1038/s41598-017-12626-7 (2017). 10.1038/s41598-017-12626-7 PMC5626686 28974736

[89] Thuillard, S. & Dan-Glauser, E. Efficiency of illusory choice used as a variant of situation selection for regulating emotions: reduction of positive experience but preservation of physiological downregulation. *Appl. Psychophysiol. Biofeedback*. **46**, 115–132. 10.1007/s10484-020-09484-x (2021). 32770450 10.1007/s10484-020-09484-x PMC7878267

[90] Thuillard, S. & Dan-Glauser, E. The simultaneous use of emotional suppression and situation selection to regulate emotions incrementally favors physiological responses. *BMC Psychol.* **8**10.1186/s40359-020-00495-1 (2020). 10.1186/s40359-020-00495-1 PMC7731633 33308297

[91] Thuillard, S. & Dan-Glauser, E. Situation selection for the regulation of emotion responses: Non-meaningful choice options retain partial physiological regulatory impact. *Int. J. Psychophysiol.* **162**, 130–144. 10.1016/j.ijpsycho.2021.02.009 (2021). 33581171 10.1016/j.ijpsycho.2021.02.009

[92] Trentini, E. & Dan-Glauser, E. Which emotion regulation strategy is efficient for whom? Reappraisal and suppression efficiency for adaptive and maladaptive personality profiles. *J. Personal. Adv. Online Publ.* (2024). 10.1111/jopy.12948 PMC11891968 38801169

[93] Trentini, E. & Dan-Glauser, E. Use of a difference index approach to analyze the early dynamic efficiency of reappraisal and suppression. *Methods Psychol.* **8**10.1016/j.metip.2023.100112 (2023).

[94] Trentini, E. & Dan-Glauser, E. Which emotion regulation strategy is efficient for whom? Reappraisal and suppression efficiency for adaptive and maladaptive personality profiles. *J. Pers.* **93**, 463–488. 10.1111/jopy.12948 (2025). 38801169 10.1111/jopy.12948 PMC11891968

[95] Salamon, N., Augsburger, N. & Dan-Glauser, E. Benefits of planning as an emotion regulation strategy in obsessions and compulsions (OC): implication of the perfectionism level in the planning-OC relationship. *J. Affect. Disord. Rep.* **14** (2023).

[96] Bradley, B. et al. Emotion dysregulation and negative affect: association with psychiatric symptoms. *J. Clin. Psychiatry*. **72**, 6427 (2011). 10.4088/JCP.10m06409blu PMC4605672 21658350

[97] Saxena, P., Dubey, A. & Pandey, R. Role of emotion regulation difficulties in predicting mental health and well-being. *SIS J. Proj. Psychol. Mental Health*. **18**, 147–155 (2011).

[98] Paica, I., Martinsone, K. & Taube, M. in *International Scientific Conference on Society, Integration, Education.* 145–154 (2020).

[99] Wu, C. et al. Emotion regulation difficulties in depression and anxiety: evidence from the dynamics of strategy use and daily affect. *J. Context. Behav. Sci.* **33**. 10.1016/j.jcbs.2024.100781 (2024).

[100] Shukla, M. & Pandey, R. Identifying the transdiagnostic and unique domains of emotion regulation difficulties in subclinical conditions of anxiety and co-occurring anxiety-depression. *Curr. Psychol.* **40**, 2896–2909. 10.1007/s12144-019-00224-x (2021).

[101] Pilkington, P. D., Karantzas, G. C., Faustino, B. & Pizarro-Campagna, E. Early maladaptive schemas, emotion regulation difficulties and alexithymia: A systematic review and meta-analysis. *Clin. Psychol. Psychother.* **31**. 10.1002/cpp.2914 (2024). 10.1002/cpp.2914 37735142

[102] Preece, D. A. et al. Alexithymia and emotion regulation. *J. Affect. Disord.* **324**, 232–238 (2023). 36566943 10.1016/j.jad.2022.12.065

[103] Pandey, R., Saxena, P. & Dubey, A. Emotion regulation difficulties in alexithymia and mental health. *Europe’s J. Psychol.* **7**, 604–623 (2011).

[104] Watson, D., Clark, A. L. & Tellengen, D. Development and validation of brief measure of positive and negative affect: the PANAS scales. *J. Personal. Soc. Psychol.* **54**, 1063–1070 (1988). 10.1037//0022-3514.54.6.1063 3397865

[105] Kroenke, K., Spitzer, R. L., Williams, J. B. W. & Löwe, B. An ultra-brief screening scale for anxiety and depression: the PHQ–4. *Psychosomatics* **50**, 613–621. 10.1016/S0033-3182(09)70864-3 (2009). 19996233 10.1176/appi.psy.50.6.613

[106] Kroenke, K., Spitzer, R. L. & Williams, J. B. W. The PHQ-9: validity of a brief depression severity measure. *J. Gen. Intern. Med.* **16**, 606–613. 10.1046/j.1525-1497.2001.016009606.x (2001). 11556941 10.1046/j.1525-1497.2001.016009606.x PMC1495268

[107] Spitzer, R. L., Kroenke, K., Williams, J. B. & Löwe, B. A brief measure for assessing generalized anxiety disorder: the GAD-7. *Arch. Intern. Med.* **166**, 1092–1097 (2006). 16717171 10.1001/archinte.166.10.1092

[108] Bouvard, M. *Questionnaire et Echelles d’évaluation de la Personnalité* (Edition Masson, 1999).

[109] The Jamovi Project. The jamovi project (version 2.4.8) (2024).

[110] Cheung, G. W. & Rensvold, R. B. Evaluating Goodness-of-Fit indexes for testing measurement invariance. *Struct. Equ. Model. Multidiscip. J.* **9**, 233–255. 10.1207/S15328007SEM0902_5 (2002).

[111] Chen, F. F. Sensitivity of goodness of fit indexes to lack of measurement invariance. *Struct. Equ. Model. Multidiscip. J.* **14**, 464–504. 10.1080/10705510701301834 (2007).

[112] Hu, J. The integration of EFA and CFA: one method of evaluating the construct validity. *Global J. Human-Social Sci. Res.* **15**, 15–19 (2015).

