# Supplementary material for: Validation of a French version of the DERS-SF and creation of a super short form (DERS-F SSF) for measuring emotion regulation difficulties
Source: Sci Rep. 2025 Nov 19;15:40702. doi: 10.1038/s41598-025-24281-4 (PMC12630587; doi:10.1038/s41598-025-24281-4)
Supplement: Supplementary file 1 — Supplementary Material 1 [file 41598_2025_24281_MOESM1_ESM.docx]

**Supplementary Table 1**

**Measurement invariance across gender for the DERS scales**

| **Version** | **Model** | **χ² (df)** | **CFI** | **ΔCFI** | **RMSEA [90% CI]** | **ΔRMSEA** | **SRMR** | **ΔSRMR** | **TLI** |
| --- | --- | --- | --- | --- | --- | --- | --- | --- | --- |
| **DERS-36** | Configural | 7930.23 (1158) | .881 | – | .066 [0.065–0.068] | – | .083 | – | .871 |
|  | Metric | 7992.08 (1188) | .881 | .000 | .066 [0.064–0.067] | .000 | .084 | +.001 | .874 |
|  | Scalar | 8452.60 (1218) | .873 | –.008 | .067 [0.066–0.068] | +.001 | .083 | –.001 | .869 |
|  |  |  |  |  |  |  |  |  |  |
| **DERS-18 Standalone** | Configural | 1101.79 (240) | .879 | – | .094 [0.089–0.100] | – | .084 | – | .846 |
|  | Metric | 1119.40 (252) | .879 | .000 | .092 [0.087–0.098] | –.002 | .085 | +.001 | .852 |
|  | Scalar | 1140.96 (264) | .877 | –.002 | .091 [0.085–0.096] | –.001 | .085 | .000 | .858 |
|  |  |  |  |  |  |  |  |  |  |
| **DERS-18 Extracted** | Configural | 1192.16 (240) | .960 | – | .055 [0.052–0.058] | – | .059 | – | .949 |
|  | Metric | 1216.19 (252) | .960 | .000 | .054 [0.051–0.057] | –.001 | .060 | +.001 | .951 |
|  | Scalar | 1274.84 (264) | .958 | –.002 | .054 [0.051–0.057] | .000 | .060 | .000 | .951 |
|  |  |  |  |  |  |  |  |  |  |
| **DERS-18 Full** | Configural | 1868.37 (240) | .947 | – | .063 [0.060–0.065] | – | .064 | – | .932 |
|  | Metric | 1890.61 (252) | .946 | –.001 | .061 [0.059–0.064] | –.002 | .064 | .000 | .935 |
|  | Scalar | 1956.32 (264) | .945 | –.001 | .061 [0.058–0.063] | .000 | .064 | .000 | .936 |

*Note.* Interpretation had the following thresholds: when moving from a less constrained model (configural → metric → scalar) to a more constrained model invariance is supported when ΔCFI ≤ .010, ΔRMSEA ≤ .015 ΔSRMR ≤ .030 for metric, ΔSRMR ≤ .015 for scalar (Chen, 2007, Cheung & Rensvold, 2002)
